# Effects of foliage-applied exogenous *γ*-aminobutyric acid on seedling growth of two rice varieties under salt stress

**DOI:** 10.1371/journal.pone.0281846

**Published:** 2023-02-23

**Authors:** Di Feng, Qian Gao, Xiaoan Sun, Songrui Ning, Na Qi, Zetian Hua, Jingchun Tang

**Affiliations:** 1 Weifang University of Science and Technology, Shouguang, Shandong, China; 2 Tianjin Tianlong Technology Corporation Limited, Tianjin, China; 3 College of Environmental Science and Engineering, Nankai University, Tianjin, China; 4 Institute of Plant Protection, Chinese Academy of Agricultural Sciences, Beijing, China; 5 State Key Laboratory of Eco-hydraulics in Northwest Arid Region of China, Xi’an University of Technology, Xi’an, Shaanxi, China; Bangabandhu Sheikh Mujibur Rahman Agricultural University, BANGLADESH

## Abstract

Exogenous *γ*-aminobutyric acid (GABA) has been used and regarded as a potential enhancer for plant resistance against various biotic or abiotic attackers in the crop production, especially as a promising alleviator against salt stress. In order to determine whether GABA is truly effective in promoting rice resistance under a certain level of salt stress or not and to evaluate its effect on the growth and some physiological responses of two Japonica rice varieties under salt stress. 3-leaf rice seedlings germinated from seeds were cultivated in a separate hydroponic cup with a nutrient solution that was salinized with 0, 25, 50, or 75 mmol K^+^ of NaCl. A 4 mmol L^−1^ GABA solution or water were sprayed onto leaves once a day for 8 days prior to an assessment of the seedling growth, the growth indices, root activities and three antioxidant enzyme activities in leaves were measured. Data analyses indicated that as the salt concentration increased, the plant height and the leaf area of both rice varieties decreased, while the dead leaf rate, weight ratio of the dry- and fresh-roots, superoxide dismutase (SOD) and peroxidase (POD) activities increased. Under the same saline conditions, the root activities and the leaf ascorbate peroxidase (APX) activity were enhanced at a low NaCl concentration but reduced when the salt concentration was high. A foliar application of GABA daily on both rice varieties for over a week under 3 different salinized treatments as compared with the corresponding treatments sprayed with water resulted in an enhanced effect on plant height increment by 1.7-32.4%, a reduction of dead leaf rate by 1.6-36.4%, a decline of root dry weight by 9.3-30.9% respectively, and an increment in root activities by 8.1-114.5%, and POD, SOD and APX enzyme activities increased by 5.0-33.3%, 4.1-18.5%, and 7.2-64.4% respectively. However, two rice varieties showed a significant difference in response to various salinized levels. Overall results of this study demonstrate that the application of exogenous GABA on the leaves of rice seedlings under salt stress has improved rice salt tolerance, which should provide a sufficient information for ultimately making it possible to grow rice in salinized soil.

## Introduction

Salt stress is considered as one of common and devastating abiotic stresses that reportedly limits the growth, productivity and cultivation of plants and crops [1, 2]. Salt stress is mainly induced by excessive sodium (Na^+^) and chloride (Cl^−^) ions [3, 4], affecting the physiological and biochemical processes in crops from the seed germination to the crop harvest due to ionic, osmotic and oxidative fluctuations [5–7]. Rice (*Oryza* *sativa* L.) serving as an essential food for more than half of the world’s population is one of the salinity-sensitive paddy crops [2, 8, 9]. Because a paddy field farming provides sufficient water that is ideal and required to leach excessive salts in saline soil, rice has usually been chosen as the preferred crop for agricultural utilization of saline alkali lands. Previous studies have shown that rice is capable of responding to salt stress through a series of salt tolerant mechanisms to some extent [3], but it can be affected, especially at the seedling stage when the electrical conductivity (EC) of the soil water exceeds 3 dS *m*^−1^ [10]. The excessive soil salinity inhibits the seed germination, reduces the bud length, slows down the root growth such as its length and number, etc. [11, 12]. More damages due to salt stress also include: 1) the inhibition of the leaf elongation and the number of tillers; 2) a prolonged tillering process; 3) a reduction of the total number of flowers; 4) a lower weight per 1,000 grains; and 5) a loss in rice yield and quality [13]. During the rice growing season, once salt damages occur, it would be extremely difficult to take any agronomic measures to reverse them. Therefore, it is practically important to develop an easy and feasible technique for this type of emergent scenario in rice production under salt stress. And, use of exogenous substances (ESs) to establish an effective and sustainable tolerance against salinity could become a potential and promising solution to alleviate crop salt stress since it is rather simple for a field application and not constrained by the growing stages and seasonal changes of a crop.

Up to the present, more than 58 ESs have been reported to play a role in alleviating plant salt stress [14]. Among them, *γ*-aminobutyric acid (GABA) is a non-protein four-carbon amino acid that is associated with various physiological responses such as the regulation of cytosolic pH, carbon fluxes in the tricarboxylic acid cycle, and nitrogen metabolism [15]. Many researchers have found that an appropriate amount of exogenous GABA could promote the plant growth, antioxidant metabolism, and transcript levels of genes encoding antioxidant enzymes so to alleviate salinity-caused oxidative damages in plants [16]. Wang et al. concluded that salt tolerance of maize seedlings was enhanced after its seeds were soaked in a 0.5 mmol L^−1^ exogenous GABA solution under a 150 or 300 mmol L^−1^ NaCl stress, likely through increase of photosynthesis, osmo-protectants and antioxidants [17]. A same alleviation of salt stress on white clover seedlings was also reported when a 1 μmol L^−1^ GABA solution was used to soak seeds under a 100 mmol L^−1^ NaCl stress, resulting in a significant increase of the Na^+^/K^+^ content and transcript levels of genes encoding Na^+^/K^+^ transportation, e.g. *HKT1*, *HKT8*, *HAL2*, *H*^+^-*ATPase* and *SOS1* [18]. Li et al. suggested that use of 0.5 mmol L^−1^ exogenous GABA in the hydroponics solution with less than 300 mmol L^−1^ NaCl in it to grow wheat seedlings could improve photosynthesis and chlorophyll fluorescence parameters, increase antioxidant enzyme activities, and reduce a malondialdehyde accumulation in leaves [19]. Similar results were reported by Wu et al. [20] who also found that 5 mmol L^−1^ exogenous GABA hydroponics plus 175 mmol L^−1^ NaCl significantly reduced Na^+^ accumulation in tomato leaves and roots by preventing a Na^+^ influx into roots and the Na^+^ movement further from roots to leaves. Kalhor et al indicated that 25 *μ* mol L^−1^ of GABA hydroponics plus 40 or 80 mmol L^−1^ NaCl significantly reduced the negative effects of salinity on lettuce growth by improving the photosystem II and reducing electrolyte leakage [21]. Zhao et al. found that when soil contained 0.15% NaCl, a spraying of 4 mmol L^−1^ exogenous GABA on rice leaves at the tillering had the most effectiveness in improving the antioxidant metabolisms and increasing the rice yield [22]. As summarized above, the effectiveness of exogenous GABA varies depending on its application methods and timing, its concentrations, plant species, crop growing stages, salt stress intensity, and duration of soil salinization. Due to the unlikelihood of growing rice in hydroponics in a field setting, it is more feasible and meaningful to carry out an experiment to determine the effect of spraying GABA on the growth of rice seedlings.

In a large scale, rice is generally grown through transplanting [23], so the seed germination and seedling production can be well managed in nursery facilities. These 3-leaf-1-bud rice seedlings are vulnerable and will encounter various adverse stresses such as salinity once they are transplanted to the paddle fields. Therefore, it would be worthwhile and beneficial to study ESs such as GABA on its potential effects on salt tolerance of rice seedlings and its sustainability through the whole rice growing season. Since there have been few reports of exogenous GABA applications on rice growth, we selected two main Japanese rice varieties ‘Tianlongyou 619’ and ‘Dajueyipin’ in North China to: 1) evaluate effects of foliage-sprayed GABA on the growth of rice seedlings; 2) reveal the GABA participated salt tolerance at various levels of salt stress and its possibly involvement in the antioxidant defensive pathway in both rice varieties. The results and conclusions derived from this study should provide some useful information for improving rice production under salt stress in the salinized paddy field.

## Materials and methods

### Materials

This experiment was carried out from July to September 2021 at the National Japonica Rice Engineering Technology Research Center (Tianjin, China). Two japonica rice varieties, ‘Tianlongyou 619’ and ‘Dajueyipin’, were provided by Tianjin Tianlong Technology Co., Ltd. GABA is provided by Shandong Shengyuan Biotechnology Co., Ltd. The seedling cultivation substrate was prepared by mixing soddy soil, perlite and vermiculite in proportion of 3:1:1 (Jinsheng, Shouguang, Shandong). The nutrient components were: organic mass 35.3%, hydrolysis nitrogen 2,531 mg kg^−1^, available phosphorus 338.9 mg kg^−1^, and available potassium 2,334 mg kg^−1^. Half-strength Kimura B nutrient solution formula [24] was used for the rice nutrient solution.

### Experimental design

#### Cultivation of seedlings

Healthy rice seeds were disinfected with 70% alcohol for 1 min, washed with distilled water, soaked for 36 h in a dark container, and then sowed in a 72-holes tray. Seedlings in the tray were grown outdoors and watered daily without fertilization before being transplanted for the experiment.

#### Hydroponic experiment

The indoor hydroponic experiment started when rice seedlings reached the 3-leaf-1-heart stage prior to tillering. Four NaCl concentrations (0, 25, 50 and 75 mmol L^−1^) were prepared in hydroponic cups (10 cm high and 8 cm in diameter) with a nutrient solution to generate a series of artificial salt stress since it contributes approximately 50% of salinity to irrigated soils [3]. To use a proper and adequate concentration of exogenous GABA, five concentrations (0, 2, 4, 8, 16 mmol L^−1^) were tested in a pilot trial for the most significant mitigating effect on alleviating salt stress, from which the 4 mmol L^−1^ GABA solution described by Sha et al. [25] was the best concentration in improving plant salt tolerance, therefore was chosen for the foliar application. Exogenous GABA was sprayed once a day on both sides of leaves until they were completely wet. The control group without a GABA application was sprayed with water instead. The salt (S) alone or salt plus GABA (SG) treatments at a zero salt and three salinized levels (0, 25, 50, and 75 mmol L^−1^) that were designated as S0, S25, S50, S75 and SG0, SG25, SG50, SG75, respectively.

There were 16 treatments in total with the experiment, 4 replications (cups) for each treatment, and 6 seedlings per cup. A nutrient solution was used to grow seedling growth and different amount of NaCl was added to prepare to generate a certain level of salt stress in the hydroponic cultivation. The hydroponic rice solutions were replaced or replenished once in two days. Indoor supplementary lighting was timed to switch on and off for 14 h per day at a full spectrum. The average temperature of the plant growth environment was 25°*C*. The experiment ended 8 days after the hydroponic experiment started (DAHE).

#### Sampling and growth assessment

1) *Seedling morphological indexes*. The plant height of all rice seedlings was measured using a ruler before and on the 8th DAHE, yellowing leaves counted on the 8th DAHE, and the dead leaf rate calculated. With each treatment, five uniform seedlings were measured for the length and width of expanded leaves using a ruler, and the leaf area was calculated using the formula proposed using Eqs (1) and (2) described by Deng and Wu [26]. After measurements, all six seedlings were divided into three groups to obtain roots and leaves for evaluation of antioxidant enzyme activity. The fresh weight of leaves was obtained through using an electronic balance with an accuracy of 0.001 g, and then fresh tissues were dried in an oven at 75°*C* to a constant dried weight for a ratio of dry and fresh weight. Fresh roots were obtained from the cultivated seedlings and their root activity was determined using an assay kit (Suzhou Keming Biotechnology Co., Ltd., China). Salt tolerance index was calculated using Eq (3).

$$\begin{array}{c} S=\text{KLD} \end{array}$$ (1)

$$\begin{array}{c} K=0.813e^{-\frac{1.2879}{X}} \end{array}$$ (2)

where L and D is the length and width of expanded leaves, respectively; K is the conversion factor; X = L/D.

$$\begin{array}{c} \text{St}=\frac{G_{i}}{G_{0}}\times 100 \end{array}$$ (3)

where St is the salt tolerance index, G_i_ is the growth indicators under salt stress, G_0_ is the growth indicators under zero salt stress.

2) *Antioxidant enzyme activity in seedling leaves*. Fully expanded true leaves (3rd leaf from the top) of the rest seedlings were cut and mixed to determine the antioxidant enzyme activity. Superoxide dismutase (SOD), peroxidase (POD) and ascorbate peroxidase (APX) activities that have been proven to be the most indicative and meaningful enzymes actively involved in responses against salt stress were measured using an assay kit (Suzhou Keming Biotechnology Co., Ltd., China), and each measurement was repeated three times.

#### Data statistics and analysis

The data was routinely processed and plotted in Office Excel 2019 software, and SPSS 17.0 (IBM, Armonk, NY, USA) was used for the analysis of significant differences and variance of multiple factors.

## Results and analysis

### Effect of GABA on the growth of rice seedlings under salt stress

**Fig 1:**
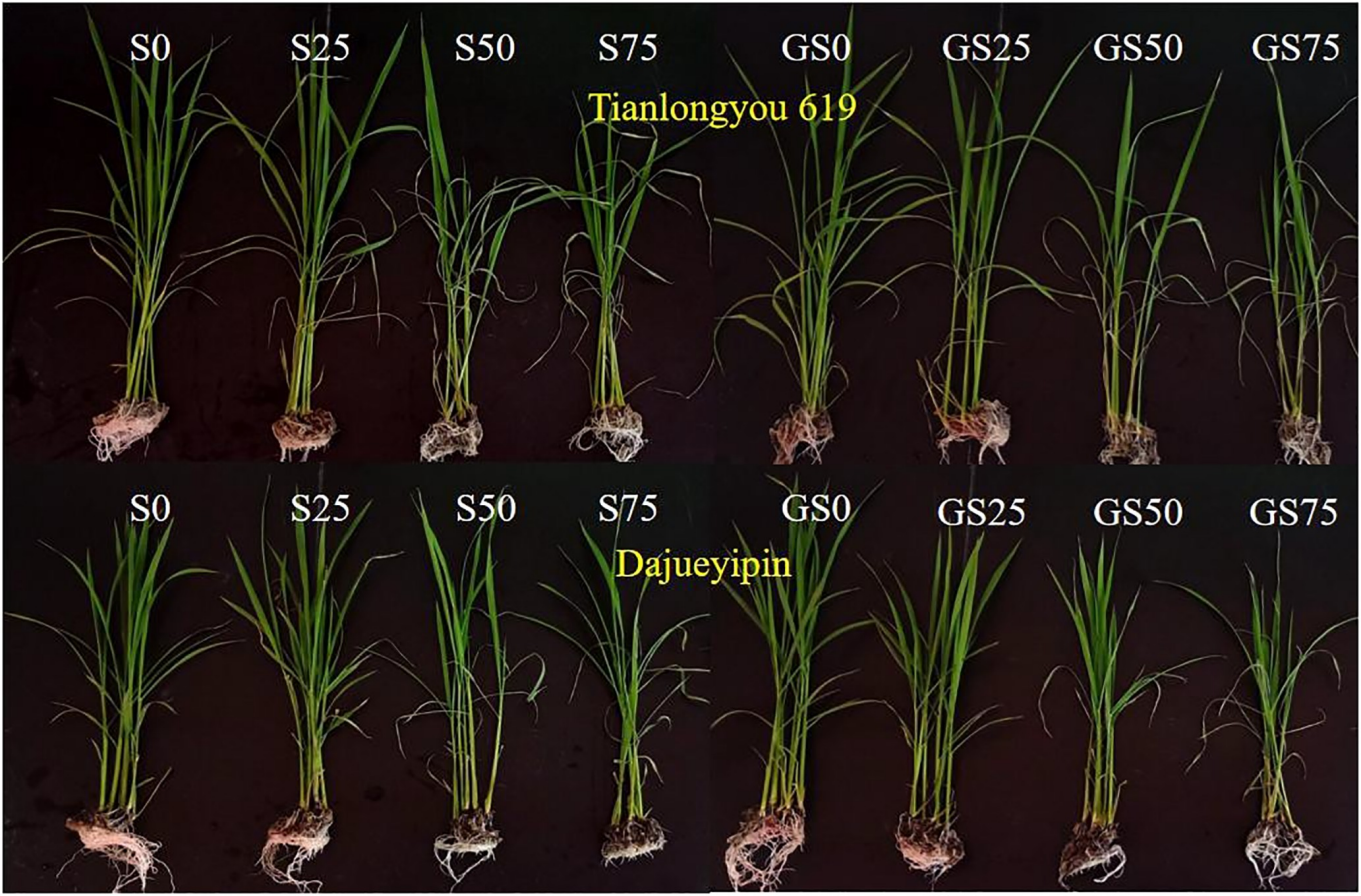
Rice seedling growth under different salt or/and GABA treatments at the end of the experiment.

**Fig 2:**
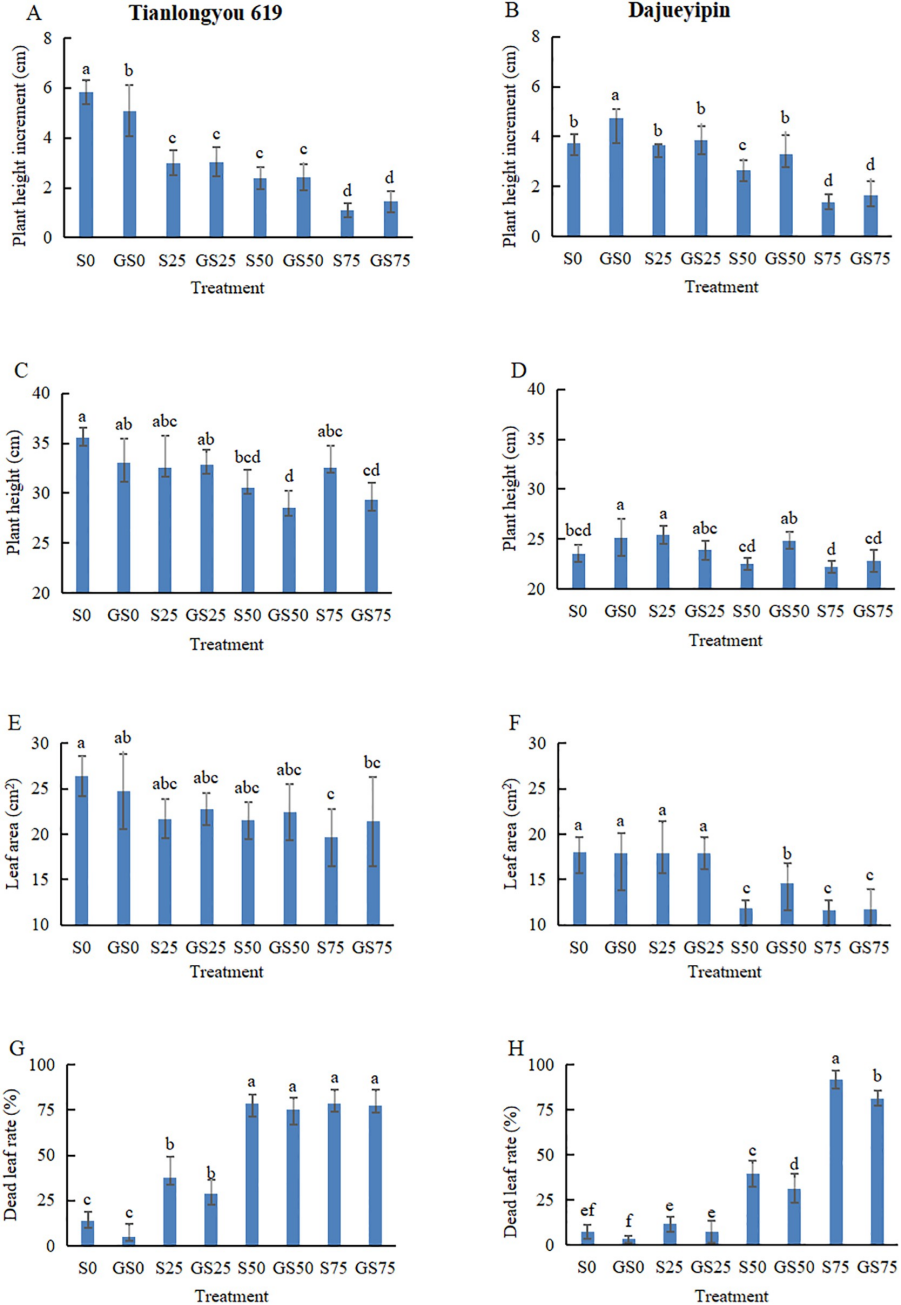
Assessment of seedling growth on two rice varieties under different salt or/and GABA treatments.

The plant height increment and the leaf area of seedlings with two rice varieties showed a downward trend while salt stress intensified (Figs 1 and 2) and the difference of plant height increment between treatments was more significant (Fig 2) than that of the leaf area. However, there was no consistent differences between treatments (*P* > 0.05) in terms of the plant height alone due to the measurements were greatly affected by the variation of the initial seedling heights. The percentage of dead leaves with all salt stressed treatments increased significantly (*P* < 0.05) in comparison with the NaCl-free treatment. As shown in Table 1, two rice varieties suffered different salt damages at various levels. When the NaCl concentration was less than 75 mmol/L, the plant height increment of ‘Tianlongyou 619’ was significantly less than ‘Dajueyipin’. With the leaf area, ‘Tianlongyou 619’ suffered more than ‘Dajueyipin’ at the 25 mmol L^−1^ NaCl concentration, but was affected less than ‘Dajueyipin’ under the 50 or 75 mmol L^−1^ NaCl stress.

**Table 1:** Salt tolerance index of aboveground growth indicators of rice seedlings.

Treatment	Tianlongyou 619		Dajueyipin	
	Plant height increment	Leaf area	Plant height increment	Leaf area
S0	100	100	100	100
S25	51.1	82.3	98.2	99.5
S50	40.7	81.6	71.6	65.7
S75	18.8	74.4	36.9	64.7

Note: Salt tolerance index is the ratio of growth indicators under salt stress to those with zero salt stress ×100.

Compared with the treatment without GABA spraying, the plant height increment of ‘Tianlongyou 619’ at the 0, 25, 50 or 75 mmol L^−1^ NaCl concentration increased by -12.9%, 1.7%, 2.3% and 32.4%, the leaf area increased by -6.2%, 4.9%, 4.3% and 9.1%, and the percentage of dead leaves decreased by 63.6%, 23.3%, 4.8% and 1.6%, respectively; while the plant height increment of ‘Dajueyipin’ at four different salt stresses showed a 27.2%, 5.7%, 23.4% and 19.4% increase with the plant height increment, a -0.2%, 0.1%, 23.9% and 0.9% rise with the leaf area, and a 57.1%, 36.4%, 21.1% and 11.4% decrease with the percentage of dead leaves, respectively. It could be seen that spraying GABA alleviated salt stress and improved the growth of rice seedlings, which was also correlated to salt tolerance of different rice varieties/varieties and the salt stress intensity. In addition, spraying GABA had a greater effect on promoting plant height growth of ‘Dajueyipin’ cultivated in the nutrient solution with less than 50 mmol L^−1^ of NaCl.

Values followed by different letters are significantly different at *P* < 0.05. Error bars represent standard deviation (± SD). S0, S25, S50, and S75 indicate that rice seedlings were cultivated in 0, 25, 50, 75 mmol L^−1^ NaCl solution, respectively, while GS0, GS25, GS50, and GS75 indicate that rice seedlings were cultured in 0, 25, 50, 75 mmol L^−1^ NaCl solution and sprayed with 4 mmol L^−1^ GABA. Same notations are used in the figures bellow.

### Effects of GABA on rice seedling roots under salt stress

**Fig 3:**
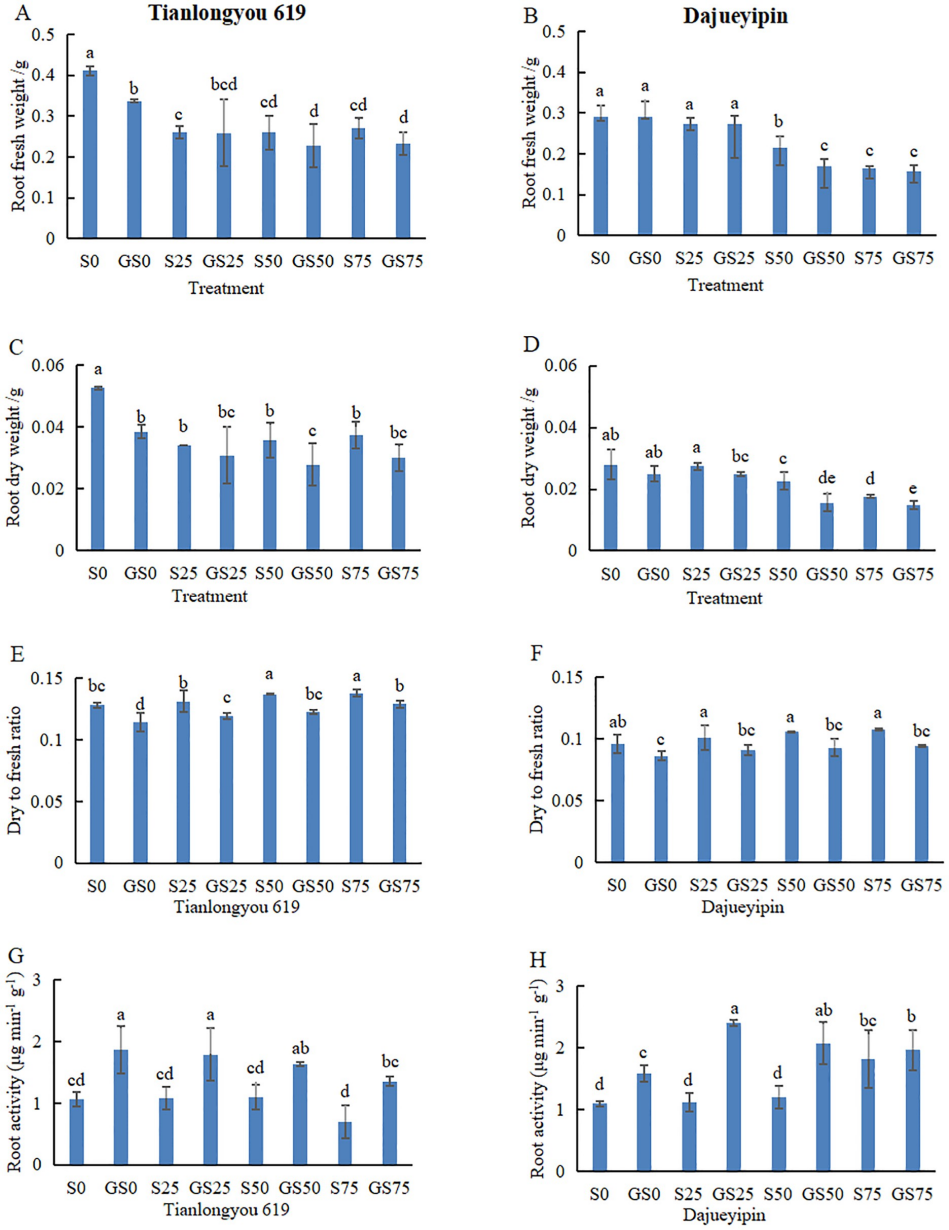
Assessment of root growth on two rice varieties under different salt or/and GABA treatments.

While salt stress intensified, the ratio of dry and fresh roots of seedlings with both rice varieties had an upward trend, but the root activity increased under less salt stress and then decreased with a higher salinity (Fig 3). However, although the root fresh and dry weight fluctuated slightly among treatments, their overall trend was decreasing alone with the increase of salinity. Under the same salt stress treatment and compared with the treatment without spraying GABA, the root fresh weight of ‘Tianlongyou’ decreased by 18.1%, 0.2%, 12.5% and 14.2%, its root dry weight reduced by 24.5%, 9.3%, 22.0% and 19.6%, but its root activity increased by 76.1%, 64.8%, 48.6% and 94.4% after GABA was sprayed on the seedlings grown in 0, 25, 50 and 75 mmol L^−1^ NaCl solutions, respectively. On the other hand, with the treatments with a GABA foliar application, the root fresh weight of ‘Dajueyipin’ decreased by 0.5%, 0.2%, 21.4%, 4.2%, its root dry weight reduced by 10.7%, 9.7%, 30.9%, 16.0%, and its root activity of the variety increased by 45.0%, 114.5%, 73.2%, 8.1%, respectively. Therefore, the foliar GABA application reduces the dry and fresh weight of rice roots and improved the root activity.

### Effects of GABA on antioxidant enzyme activities of rice seedling leaves under salt stress

**Fig 4:**
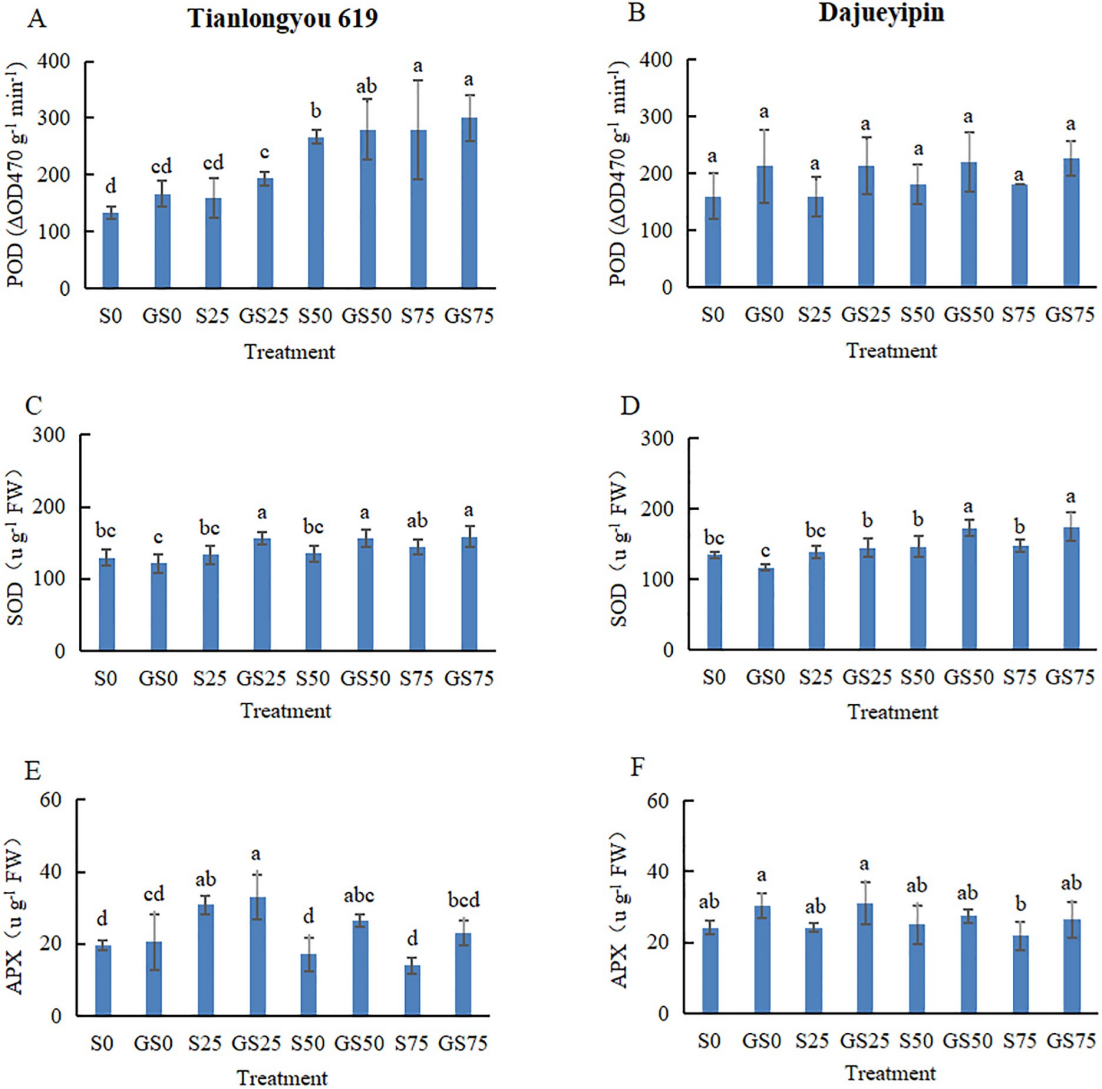
Assessment of antioxidant enzyme activities in rice leaves under different salt or/and GABA treatments.

The activity of antioxidant enzymes such as POD and SOD in leaves of rice seedling increased with all different levels of salt stress except APX that increased first at lower NaCl concentration and then decreased under high salt stress (Fig 4). In comparison with no GABA treatments at the same level of the salt stress, all three enzyme activities were enhanced with GABA foliar applications, except the SOD activity that decreased at the 0 mmol L^−1^ NaCl treatment. With each of different salt stress treatments, the POD activity in ‘Tianlongyou 619’ leaves increased by 25.0%, 20.8%, 5.0% and 7.1%, the SOD activity increased by -6.5%, 17.3%, 15.8% and 9.8%, and the APX activity increased by 4.6%, 7.2%, 54.8% and 64.4%, respectively at four different salt stress levels with the GABA treatment. The POD activity of ‘Dajueyipin’ in leaves increased by 33.3%, 33.3%, 22.2% and 25.9%, the SOD activity increased by -13.2%, 4.1%, 18.0% and 18.5%, and the APX activity increased by 25.9%, 28.0%, 9.6% and 20.6%, respectively under the same salt stress level and the GABA treatment. So, it is obvious that foliar GABA applications against salt stress facilitated the activities of a group of antioxidant enzymes in rice seedling leaves.

### Analysis of interactions of multiply factors affecting rice seedling growth

Plant height increments throughout the whole growing period and the root dry mass at the end of the experiment were used to evaluate salt stress and its alleviation through GABA applications and their interactive effect on the growth of rice seedlings. Tables 2 and 3 presented the results of one-way analysis of the variance of three factors with different ‘varieties’, ‘spraying GABA or not’, and different ‘salt stress degrees’ and their effect on the plant height increment and root dry weight of rice seedlings. It can be seen from Table 2 that, among those three factors affecting plant height increment, the main effect of different ‘salt stress degrees’ was extremely significant (*P* < 0.01), while the main effect of ‘varieties’ and ‘spraying GABA or not’ was least significant (*P* > 0.05), with which the *P* value of ‘spraying GABA or not’ was 0.055. With the two-dimensional interaction, the main effect of ‘varieties’ × ‘spray GABA’ and ‘varieties’ × ‘salt stress degrees’ were significant (*P* < 0.05), but the main effect of ‘spray GABA’ × ‘salt stress degrees’ was not significant (*P* > 0.05). With the three-dimensional interaction, the main effect of ‘varieties’ × ‘spraying GABA or not’ × ‘salt stress degrees’ was significant (*P* ≤ 0.05). Partial Eta squared (*η*^2^) value indicated the magnitude of the main or interaction effect. Therefore, from the analyses described above, the ranking of the significance of those main and interaction effect on the plant height increment should be: ‘salt stress degrees’, ‘varieties’× ‘salt stress degrees’, ‘varieties’ × ‘spraying GABA or not’ × ‘Salt stress degrees’, ‘varieties’ × ‘spraying GABA or not’.

**Table 2:** Univariate multifactor analysis of variances of the plant height increment of rice seedlings.

Source	Type III sum of squares	*df*	Mean square	*F*	*P* Significance	*η*^2^ Partial Eta squared
Corrected model	207.072a	19	10.899	48.694	0	0.939
Intercept	517.06	1	517.06	2310.205	0	0.975
Varieties	0	1	0	0.002	0.966	0
Spraying GABA or not	0.858	1	0.858	3.832	0.055	0.06
Salt stress degrees	192.236	4	48.059	214.726	0	0.935
Varieties × Spraying GABA or not	1.221	1	1.221	5.455	0.023	0.083
Varieties × Salt stress degrees	10.303	4	2.576	11.508	0	0.434
Spraying GABA or not × Salt stress degrees	0.188	4	0.047	0.21	0.932	0.014
Varieties × Spraying GABA or not × Salt stress degrees	2.265	4	0.566	2.53	0.05	0.144
Error	13.429	60	0.224			
Total	737.56	80				
Corrected total	220.5	79				
a R^2^ = 0.939 (After adjustment R^2^ = 0.920)						

**Table 3:** Univariate multifactor analysis of variances of the root dry weight of rice seedlings.

Source	Type III sum of squares	*df*	Mean square	*F*	*P* Significance	*η*^2^ Partial Eta squared
Corrected model	0.005a	19	0	31.949	0	0.938
Intercept	0.046	1	0.046	6016.594	0	0.993
Varieties	0.002	1	0.002	305.406	0	0.884
Spraying GABA or not	0	1	0	54.507	0	0.577
Salt stress degrees	0.001	4	0	41.498	0	0.806
Varieties × Spraying GABA or not	3.53 × 10^−5^	1	3.527 × 10^−5^	4.62	0.038	0.104
Varieties × Salt stress degrees	0	4	0	13.703	0	0.578
Spraying GABA or not × Salt stress degrees	9.06 × 10^−5^	4	2.265 × 10^−5^	2.967	0.031	0.229
Varieties × Spraying GABA or not X Salt stress degrees	7.5 × 10^−5^	4	1.875 × 10^−5^	2.456	0.061	0.197
Error	0	40	7.633 × 10^−6^			
Total	0.051	60				
Corrected total	0.005	59				
a R^2^ = 0.938(After adjustment R^2^ = 0.909)						

Table 3 indicated that the main effect of all three factors (‘varieties’, ‘spraying GABA or not’ and different ‘salt stress degrees’) significantly (*P* < 0.01) affected the root dry weight, while two-dimensional interactions, such as ‘varieties’ ×’spray GABA’, ‘varieties’ × ‘salt stress degrees’ and ‘spraying GABA or not’ × ‘salt stress degrees’ were significant (*P* < 0.05). With the three-dimensional interactions, the main effect of ‘varieties’ × ‘spraying GABA or not’ × ‘salt stress degrees’ was not significant (*P* > 0.05). The order of the effect of those factors and their interactions on root dry weight was ‘varieties’, ‘salt stress degrees’, ‘varieties’ × ‘salt stress degrees’, ‘spraying GABA or not’, ‘spraying GABA or not’ × ‘salt stress degrees’, and ‘varieties’ × ‘spraying GABA or not’.

The results demonstrated that variety had the greatest effect on the growth indexes of rice seedlings that was represented by root dry mass, followed by the degree of salt stress, the interaction between ‘varieties’ and ‘salt stress degrees’, and last ‘spraying GABA or not’. The least effect of those interactions belonged to the ‘varieties’, ‘salt stress degrees’ and their interactions with the ‘spraying GABA or not”. Salt stress played the most significant role in the growth of rice seedlings that was represented by the plant height increment, followed by the interaction of ‘varieties’ × ‘salt stress degrees’, the three-dimensional interaction of ‘varieties’ × ‘spraying GABA or not’ × ‘salt stress degrees’, and the interaction of ‘varieties’ × ‘spraying GABA or not’. It was obvious that foliar-applied GABA affected the growth of rice seedlings, and there were differences with the ‘varieties’ and various plant growth factors.

## Discussion

In this study, salt stress was simulated at different levels post transplanting of rice seedlings and the results derived from data analyses were consistent with previous research findings in general, suggesting that with the increase of salinity, the plant height increment, leaf area, root dry and fresh weight of the two tested rice varieties showed a downward trend. The possible underline impact of the salt impairment was likely due to inhibition of photosynthesis in rice [17, 19, 27] and thereby reduction in the accumulation of photosynthetic products. while the percentage of dead leaves increased due to leaf chlorosis caused by an excessive accumulation of Na^+^ and Cl^−^ [13, 28]. Moreover, the root dry-fresh ratio shows an upward trend possibly due to limited water absorption and less free water available in the plant. Through analyzing the factors that possibly affected the growth of rice seedlings, we found that rice varieties had the greatest impact, followed by the degree of salinity and the interaction between them on alleviation of salt stress.

Rice root activities and their changes are regarded as reliable estimators for the healthy root growth and plant salt resilience at the end of the Es treatment under salt stress. Our data analysis also indicated that rice root activities were slightly boosted first at the least salt stress and then reduced when salt concentration was higher, which was different from the findings previous reported under salt stress because a lower salt concentration (75 mmol L^−1^ NaCl solution) instead of a higher one (80 mmol L^−1^ NaCl solution) was used in a previous report [27]. A low level of salt stress does not seemingly inhibit the root activity of ‘Dajueyipin’, instead shows a significant enhancement effect under a low salt stress, while ‘Tianlongyou 619’ expresses a significant reduction of the root activity at the same level of the salt stress, suggesting a significant difference between rice varieties in their responses to the various degrees of salinity. In addition, when being under salt stress, rice tends to self-adjust and adapt to excessive salinity by increasing the activity of antioxidant enzymes to protect plant from ROS accumulation, which is considered to cause reductions in photosynthetic system efficiency [20, 29]. Our results indicated that with the increase of salt stress, POD and SOD activities in rice leaves expressed an upward trend, but APX activity did increase at lower salt stress first then reduced when the NaCl concentration, which was consistent with the results of previous studies [11, 20, 22].

Two aboveground plant growth indexes, the plant height increment and the leaf area were measured to present salt tolerance of two rice varieties, the smaller the value of salt tolerance index is, the greater the degree of salt damage would be, and vice versa. The evidence provided through this study indicates that both aboveground plant growth indexes well reflect the responses of rice seedings to different levels of salt stress even if a significant difference between two rice varieties exists in terms of their salt tolerance. However, we were unable to see the difference of plant heights among or between different salt-stressed treatments possibly due to the fact that the height of initial rice seedlings varied or/and that the duration of the experiment was not long enough so as to generate a significant difference among each individual seedlings, which might have concealed the true effect of GABA among the treatments on promoting plant growth indexed as the plant height.

Leaves are the most important vegetative tissues of plants other than roots. Once being applied on leaves, exogenous substances enter the leaf surface first by diffusing through the wax and cuticle layers and then are absorbed and utilized by mesophyll cells. This direct foliar application of GABA or other exogenous substances enables plants to absorb these potentially foreign alleviators substances more quickly, allowing them to initiate possible defensive mechanisms, to regulate the ion balance in leaf tissues, and to stabilize enzyme activities against salt stress or subsequent damages [30]. Our study also indicates that foliar spraying of exogenous substances does not only induce aboveground parts of plants to respond to and signal all biological and physiological metabolisms against salt stress, but also allow a migration of those substance to underground roots through generating a systematic resistance against an excessive salination as a whole [30]. When a salt stress occurs at any growing stages during the rice production, it would be very feasible to mitigate potential salt damages by spraying ESs on leaves. GABA alleviates the oxidative impairment to plant cells, mitigates plant physiological damage and ultimately maintains the relatively normal growth of plants by participating in the osmotic regulation, promoting the content of proline and soluble protein, reducing the ratio of sodium to potassium, and improving the activity of antioxidant enzymes [31]. Data analysis of physiological indicators with this study has shown that exogenous GABA application on rice foliage could reverse salt damages on growth of rice seedlings and that different rice varieties at their different growth stages responded differently to the various degrees of salinity. Under the same salt concentration and compared with the GABA-free treatments, the activities of antioxidant enzymes (POD, SOD and APX) in rice leaves with a GABA application under the salt stresses of 25, 50, 75 mmol L^−1^ NaCl increased to a various extent, which was consistent with the previous reports on maize [17], wheat [19], tomato [20], rice [22, 25],and other crops. While under the salt stress of the 25, 50 and 75 mmol L^−1^ NaCl treatments and in the GABA treated seedlings, the plant height increment and leaf area increased, the percentage of dead leaves decreased, the fresh and dry weight of roots decreased, and the root activity increased in general. Therefore, it is concluded that foliage-sprayed GABA can promote the growth of the aboveground parts of rice seedlings in response to salt stress, reduce the allocation of photosynthetic products to roots, and improve the root activity. There were many reports of exogenous GABA on improving the growth of aboveground plant parts [17, 19, 20, 25], but few reports of it on its reducing function on the root mass. Cheng et al. reported that the root length of white clover first increased and then decreased as the concentration of GABA was between 0 and 5 *μ* mol L^−1^ under a 100 mg L^−1^ NaCl salt stress [18]. In a petri dish cultivation setting, Kaur and Zhawar treated rice seedling for 72 h with a 100 mmol L^−1^ of a saline-alkali stress and found that the dry weight of aboveground plant parts and root of rice seedlings increased in day 5 after those seedlings were treated with 1.5 mmol L^−1^ GABA [32]. The different outcomes in terms of the fresh and dry weight of roots and aboveground parts from our study and Kaur and Zhawar’s may be due to that: 1) as a paddy crop, rice has strong tolerance against flooding and when exogenous GABA is used to alleviate salt stress, it does not need to allocate more photosynthetic products for roots to grow and function normally; 2) that Kaur and Zhawar [32] used seedlings at the bud stage during which rapidly elongated rice radicle and hypocotyl used required nutrients from seeds, which did not involve in the process of allocating photosynthetic products. In addition, exogenous GABA, as an amino acid nitrogen fertilizer, would promote the growth of shoot and root of bud seedling [33]. Although this study indicates that the foliar application of GABA is direct and effective in deliver exogenous substances but more need to be done to determine the optimal GABA application protocol, including the formulation, total amount of usage, amount and concentration of a single use, timing, spraying intervals, and application methods, etc.

## Conclusion

Rice seedlings with 3 leaves and 1 heart was cultivated in a hydroponic setting under four levels of salt stress and a 4 mmol L^−1^ exogenous GABA solution was sprayed on foliage of rice seedlings daily for 8 days. Our results derived from assessments of plant growth indexes, root activities, and antioxidant enzymes show that both salt stress and rice variety have primarily affected the growth of rice seedlings and the foliar-applied GABA has improved the activity of antioxidant enzymes and the root function of both tested rice varieties, thereby has enhanced salt tolerance. However, different rice varieties may response to salt stress differently and the effect of foliar-applied GABA may vary accordingly in mitigating the damages caused by excessive salinity. This study also confirms that GABA application on leaves of rice seedlings has enhance the movement of photosynthetic products within the aboveground plant tissue. Finally, the concentration and foliage delivery method of exogenous GABA are adequate to be used in cultivation of rice seedings in nursery to develop a potential tolerance against salt stress, especially at a mild or a mild-to-severe level. A further field trial or prolonged duration of rice growing would be useful to evaluate the effect of exogenous GABA on salt stress alleviation and to determine if the alleviating effect lasts throughout the whole rice growing season or is just temporary.
